# Spectacle wearing in children randomised to ready-made or custom spectacles, and potential cost savings to programmes: study protocol for a randomised controlled trial

**DOI:** 10.1186/s13063-016-1167-x

**Published:** 2016-01-19

**Authors:** Priya Morjaria, Kaushik Murali, Jennifer Evans, Clare Gilbert

**Affiliations:** International Centre for Eye Health (ICEH), Clinical Research Department, London School of Hygiene & Tropical Medicine, Keppel Street, London, WC1E 7HT UK; Sankara Eye Hospital, Varthur Main Road, Marthahalli, Bangalore, Karnataka 560037 India

**Keywords:** Uncorrected refractive errors, Children, School eye health, India, Spectacle wearing rate, Ready-made spectacles, Randomised clinical trial

## Abstract

**Background:**

Uncorrected refractive errors are the commonest cause of visual impairment in children, with myopia being the most frequent type. Myopia usually starts around 9 years of age and progresses throughout adolescence. Hyperopia usually affects younger children, and astigmatism affects all age groups. Many children have a combination of myopia and astigmatism. To correct refractive errors, the type and degree of refractive error are measured and appropriate corrective lenses prescribed and dispensed in the spectacle frame of choice. Custom spectacles (that is, with the correction specifically required for that individual) are required if astigmatism is present, and/or the refractive error differs between eyes. Spectacles without astigmatic correction and where the refractive error is the same in both eyes are straightforward to dispense. These are known as ’ready-made’ spectacles. High-quality spectacles of this type can be produced in high volume at an extremely low cost. Although spectacle correction improves visual function, a high proportion of children do not wear their spectacles for a variety of reasons. The aim of this study is to compare spectacle wear at 3–4 months amongst school children aged 11 to 15 years who have significant, simple uncorrected refractive error randomised to ready-made or custom spectacles of equivalent quality, and to evaluate cost savings to programmes. The study will take place in urban and semi-urban government schools in Bangalore, India. The hypothesis is that similar proportions of children randomised to ready-made or custom spectacles will be wearing their spectacles at 3–4 months.

**Methods/design:**

The trial is a randomised, non-inferiority, double masked clinical trial of children with simple uncorrected refractive errors. After screening, children will be randomised to ready-made or custom spectacles. Children will choose their preferred frame design. After 3–4 months the children will be followed up to assess spectacle wear.

**Discussion:**

Ready-made spectacles have benefits for providers as well as parents and children, as a wide range of prescriptions and frame types can be taken to schools and dispensed immediately. In contrast, custom spectacles have to be individually made up in optical laboratories, and taken back to the school and given to the correct child.

**Trial registration:**

ISRCTN14715120 (Controlled-Trials.com)

Date registered: 04 February 2015

## Background

Uncorrected refractive errors are the commonest cause of visual loss in children [1]. Myopia (short-sightedness) is the commonest form; it usually starts around the age of 9 to 10 years, progressing in severity throughout adolescence. Hypermetropia (long-sightedness), which is more common in younger children, usually resolves by around the age of 10 years. Astigmatism (distorted vision, measured in cylinders) affects all age groups and does not change over time. Myopia is far more common in Asian children, particularly in Southeast Asia. Many children with myopia also have some degree of astigmatism, and one of the standard ways of reporting refractive error is to use the ’spherical equivalent’, which is calculated as the sphere plus 0.5 x the cylinder, in dioptres (D). Refractive errors can also differ between eyes (anisometropia).

**Fig. 1:**
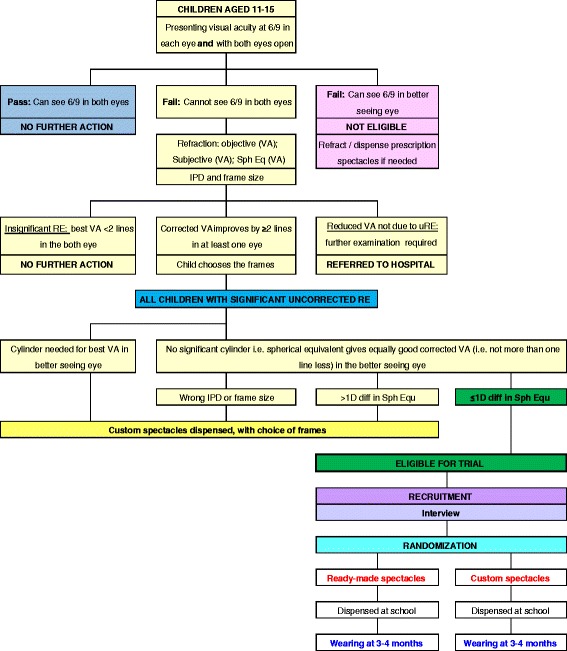
Randomisation flow chart of activities. Flow chart shows a child’s journey and the activities involved from screening to deciding whether they are eligible for recruitment, then randomisation and follow-up

Correcting refractive errors requires the following steps: measuring visual acuity in each eye without any form of correction, followed by measurement of the type and degree of refractive errors in each eye, which can be done clinically (by retinoscopy) or by an automated refractometer. The next step is to use the findings to assess which corrective lenses give the best visual acuity in each eye, which are then prescribed. The next step is to dispense the spectacles, which entails ensuring that the optical centres of the lenses required align with the visual axis of each eye when mounted in the spectacle frames of choice. If an individual has astigmatism, the axis of the cylinder in the lens must align accurately with that of the eye. Custom spectacles are needed if astigmatism is present and/or the refractive error differs between eyes. Spectacles without astigmatic correction and where the refractive error is the same in both eyes (simple refractive error) are much more straightforward to dispense. Indeed, high-quality spectacles without astigmatic correction with a range of spherical powers (the same in each eye) are being mass produced at extremely low cost (0.5US$). Several different frame sizes are also available, allowing for variation in the distance between the visual axis in different age groups, gender and populations. These spectacles are called ’ready-made’ or ’off-the-shelf’ spectacles. From a programmatic perspective, prescribing ready-made spectacles has benefits for providers as well as parents and children, as a supply of ready-made spectacles with a wide range of prescriptions and frame types can be taken to the school and dispensed immediately. In contrast, custom spectacles have to be individually made up in optical laboratories, marked with the child’s name, and the spectacles taken back to the school and given to the correct child.

The prevalence of uncorrected refractive errors in children varies by country and by urban/rural location, for example, in India [2–4]. In one study in rural India, 4.1 % of children aged 7–15 years were myopic, and 61 % of visual impairment was due to uncorrected refractive errors [2]. In an urban Indian setting 7.4 % of children aged 5–15 years were myopic and 82 % of visual impairment was due to uncorrected refractive errors. In both studies older children had a higher prevalence of uncorrected refractive error than younger children [4]. Global estimates indicate that 13 million children have visual impairment due to uncorrected refractive errors [5]. A recent study in China provides evidence of what might be anticipated, that academic performance improves with correction of refractive error in children [6]. Studies have also highlighted that correcting refractive error is highly cost effective [7] and improves visual function and quality of life. These findings add impetus to the need for the inclusion of eye health into school health initiatives, which are being supported and scaled up by Ministries of Health and Education, the World Bank, WHO, UNESCO, UNICEF and the Partnership for Child Development. India has had a programme to detect and treat uncorrected refractive errors in school children since 1994 [8].

Approaches being used to detect and correct uncorrected refractive errors in children are not standardised, and spectacle wearing rates can be very low in all settings [5]. For example, in Native American students in the USA, only 32 % of children given two pairs of free spectacles wore their spectacles [9]. Similar findings have been reported from rural areas near Delhi where only 29.4 % of children wore their spectacles [10]. Spectacle wear is higher in children with more severe uncorrected refractive errors [9] and in girls [11]. In another study in India only 30 % of children dispensed spectacles were wearing them at 6–12 months. Spectacle wearing was higher amongst girls, those with higher refractive errors and poor uncorrected visual acuity, and those whose fathers were better educated [12].

Only four trials have assessed the impact of interventions to increase spectacle wear in children, three being in low/middle income countries. One compared spectacle wear at 3–6 months in school children in Tanzania who were randomised to free spectacles or a prescription. Spectacle wear was significantly higher amongst those given free spectacles (47 % versus 26 % respectively, *p* = 0.05) [13]. In a trial in China, children were randomised to attend or not attend a health education session. Children in the health education group actually had lower rates of spectacle wear at follow-up than the controls [14]. In another study in China, a health education DVD shown to parents, teachers and children increased self-reported wear but not observed wear [6].

One study has addressed the utility of ready-made spectacles in Chinese school children. In this study children with high degrees of astigmatism, anisometropia or eye disease (8 %) were excluded and the remainder were randomised to ready-made spectacles or custom spectacles regardless of the extent to which correction improved their visual acuity. The study was powered to detect at 15 % difference in spectacle wear, but at follow-up one month later spectacle wear was similar in both groups (47 % in the ready-made spectacles group and 52 % in the custom spectacles group (*p* = 0.23) [15].

Despite spectacle correction improving visual function [16], children do not wear their spectacles for a variety of reasons, such as no perceived benefit [17], loss or breakage [18–21], misconceptions that spectacles will make their vision worse [11, 13, 22], parental disapproval [10, 15], being teased [13, 15, 18, 21–23] and forgetfulness [14, 15, 19, 21]. In a recent Indian study [12], reasons for not wearing spectacles included being teased (19.8 %), the spectacles were broken (17.4 %) or lost (9.3 %), and the child did not like their spectacles (12 %). There is also evidence that the degree of visual impairment also influences spectacle wear. For example, in the Tanzanian trial outlined above, increasing myopia was an independent predictor of spectacle wear. In a recent study in Bangalore, designed to assess different visual acuity screening cut-offs, children wearing their spectacles at 3–4 months also had higher degrees of myopia than those who were not (mean spherical equivalent in the better seeing eye −3.50 D, range −1.75 to −9.00 D versus mean −2.50 D range −0.75 to −2.25 D respectively) (*p* = 0.001) (unpublished data).

### Purpose

The purpose of this trial is to compare spectacle wear at 3 to 4 months in school children aged 11 to 15 years with significant simple uncorrected refractive errors who are randomised to ready-made spectacles or custom spectacles, and to evaluate the potential cost savings to programmes.

The hypothesis is that similar proportions of children randomised to ready-made spectacles or custom spectacles will be wearing their spectacles at 3–4 months.

### Pilot study December 2014

A pilot study was undertaken in non-trial schools to test all aspects of the trial and to provide data for the sample size calculation, including the proportion of children with uncorrected refractive errors who would be eligible for ready-made spectacles.

## Methods/design

The trial is a randomised, non-inferiority, double masked clinical trial of children with simple uncorrected refractive errors. A non-inferiority design was chosen, as the benefits of ready-made spectacles are the considerably lower cost and ease of dispensing, both of which have the potential to increase the efficiency and cost effectiveness of programmes. As millions of children are affected by uncorrected refractive errors, the lower cost of ready-made spectacles also has the potential to increase coverage of school-based programmes. Under these circumstances a slightly lower acceptance of ready-made spectacles, measured by spectacle wearing, might be acceptable. The non-inferiority margin of 10 % was chosen to balance the considerations of efficacy and secondary benefits. The allocation ratio is approximately 1:1.

### Study setting

The trial is being undertaken in government middle and secondary schools in urban and peri-urban areas in and around Bangalore, Karnataka state, India. The trial is coordinated by Sankara Eye Hospital, Bangalore. The field team consists of optometrists, dispensing opticians, field workers and ophthalmologists, all of who are members of staff at the Sankara Eye Hospital. Training, quality assurance and oversight of data collection are being provided by staff at the International Centre for Eye Health, London School of Hygiene & Tropical Medicine.

### Participant eligibility

#### Inclusion criteria

For a student to be eligible for recruitment, he/she must be aged 11–15 years, be present at school at the time of screening, and meet all the following criteria: a) presenting visual acuity (with spectacles if usually worn) of less than 6/9 in both eyes, b) visual acuity with full correction improves in the better seeing eye by two or more lines, c) the spherical equivalent corrects the visual acuity to not more than one line less than best corrected visual acuity with a full prescription in the better eye, d) the difference between the spherical equivalent of the right and left eyes is not more than 1 D, e) the inter-pupillary distance (IPD) matches that of ready-made spectacle frames available (54 to 62 mm) and f) spectacle frames are of acceptable size and fit. Parents must consent for their child to take part in the study.

#### Exclusion criteria

The following children are not being recruited: those with other causes of visual loss or whose visual acuity does not improve by two lines or more with spherical equivalent lenses; there is more than 1 D of anisometropia or parents do not consent. All these children are being dispensed custom spectacles and are not recruited to the trial.

### Eligibility of those performing interventions

All refractions, prescribing and dispensing are being undertaken by fully qualified optometrists, including the lead investigator.

### Identification of potential participants and recruitment

In the schools selected for the trial, trained field workers measure visual acuity at the 6/9 level in each eye and with both eyes open, with spectacles if the child usually wears them. A LogMAR visual acuity chart in an illuminated cabinet is being used at the recommended test distance of 6 metres to overcome variable illumination in the classrooms. Children who pass the screening test are given a green card and sent to another field worker who registers their age and gender.

All children who fail screening undergo objective and subjective refraction by an optometrist. The following information is being recorded: objective refractive error and corrected visual acuity in each eye; subjective refractive error and best corrected visual acuity in each eye. The spherical equivalent is calculated for each eye, and visual acuities are measured and recorded for each eye using the spherical equivalent. An optometrist then decides whether the child is eligible for recruitment. All children requiring spectacles, whether eligible for the trial or not, are allowed to select the frames they prefer from a range of coloured plastic or metallic frames. The type of frame and the frame size needed are recorded.

All eligible children are given an information sheet and consent form for their parents to sign which they return to the school. A trained field worker goes through an assent form with each child. Each child is allocated a unique study number and randomised to ready-made spectacles or custom spectacles. All those recruited are given a red ID card that contains their name, their father’s name, a mobile telephone number and study ID. Children are asked to give the card to their class teacher so they can be identified when the trial spectacles are delivered a few weeks later, to ensure that each child receives the correct spectacles.

Children not eligible are either given a green card, if spectacles are not required, or a red card if they need custom spectacles or referral to Sankara Eye Hospital for assessment of other eye conditions. These findings are recorded by a field worker.

In both arms of the trial the same procedures are followed, including the day on which spectacles are delivered to the school. Before giving each child their spectacles, their identity is confirmed by a field worker using the red card issued at the time of recruitment. Corrected visual acuity with the new spectacles is also measured in each eye. Children not eligible for the trial who require custom spectacles also receive their spectacles at the same time.

Children, parents/carers, and teachers are not aware which type of spectacles (ready-made spectacles or custom spectacles) each child receives (Fig. 1).

### Sample size calculation

Parameters used in the sample size calculation include a significance level of 0.05, 95 % confidence interval, 90 % power and 1:1 allocation. The trial is powered to detect a non-inferiority margin (∆) of 10 %. No increase has been added for loss to follow-up, as all eligible children present on the day of the visit are being recruited, and high response rates are anticipated based on previous experience. Calculations, which were one sided, were performed using a web-based sample size calculation programme (Sealed Envelope, https://www.sealedenvelope.com/power/binary-noninferior/, accessed 31 October 2014). A sample of 240–260 eligible children will be required in each arm of the trial. The prevalence of uncorrected refractive errors in the earlier study in Bangalore was 4 %. Assuming approximately a third would not be eligible for ready-made spectacles, the effective prevalence would be 2.6 %; therefore, 20,000 children in 200 schools would need to be screened.

### Randomisation

The random allocation has been stratified by school. An epidemiologist away from the study site generated the allocation schedule in Excel using the *rand between* function, using block randomisation with variable block sizes. Two pre-printed adhesive labels were placed in opaque envelopes, which were sealed and stamped in London and Bangalore by persons not involved in the trial. For each child recruited, the optometrist opens the next envelope in sequence to see to which arm of the trial the child has been allocated. One label has the unique study ID number and code for ready-made spectacles or custom spectacles which is adhered to the child’s data collection form. The other label, which only has the unique study ID, is adhered to the red card issued to the child.

### Primary outcome

The primary outcome is the proportion of children who are wearing their spectacles at an unannounced visit to the school 3 to 4 months after delivery of the spectacles. A field worker, masked to the allocation arm, assesses spectacle wear using categories described by Wedner [13]. Categories 1 or 2 below are used to define spectacle wearing, and categories 3 or 4 as non-spectacle wearing:

1. wearing the spectacles at the time of the unannounced visit
2. not wearing the spectacles at the time of the visit but have them at school
3. not wearing the spectacles at the time of the visit but said they are at home
4. not wearing the spectacles at the time of the visit as they are broken or lost

Children categorised as non-spectacle wearing are given an opportunity to give two reasons. The field worker asks the child the reason and this is coded and recorded on the follow-up data collection form.

Fieldwork has been planned such that the initial assessment, delivery of spectacles and follow-up 3 to 4 months later do not coincide with school examination periods, long school holidays, or the end of the school year when children may leave school.

### Data management

All field staff have undergone rigorous training, including inter-observer agreement studies for visual acuity measurement and refraction, and instruction on how to record data.

Two password protected databases have been created in Epidata and Excel, one for the primary outcome data and the other for all other data. Consistency and range checks have been built in. Data are double entered by the lead investigator as soon as possible after recruitment to monitor recruitment. During the trial all data recording forms are kept in a locked cupboard or filing cabinet in Sankara Eye Hospital and photocopies made and transferred to London for data cleaning, where they are again stored in a locked filing cabinet.

### Data analyses

Analysis will be in the groups to which the children were randomly allocated. We expect all children will have been given the correct spectacles. The randomisation code will only be broken once the analysis has been completed. We do not plan any subgroup analysis.

#### Comparability of the intervention and comparator groups

To assess comparability of the two groups, characteristics of children in the intervention and comparator arms will be compared by age, gender, degree of uncorrected refractive errors, presenting visual acuity in the better eye, peri-urban/urban school and whether they previously wore spectacles which required replacement.

#### Primary analysis

The proportion of children wearing or having their spectacles with them at school at 3 to 4 months will be compared between the intervention and comparator arms, using the risk difference with 95 % confidence intervals.

We will also calculate and present the risk ratio with 95 % confidence intervals.

#### Cost savings to programmes

Analysis of cost savings to programmes of ready-made spectacles will only be undertaken if analysis of the primary outcome demonstrates non-inferiority. The unit cost of ready-made spectacles (Cost_Ready-made spectacles_) and custom spectacles (Cost_custom spectacles_) will be calculated. The cost of dispensing spectacles to the two groups of children in the study will be determined as follows:

- A = not eligible for the trial and dispensed custom spectacles
- B = eligible for randomisation, that is, suitable for ready-made spectacles
- The cost of programmes without ready-made spectacles
  $$ \mathrm{C}\mathrm{o}\mathrm{s}{\mathrm{t}}^{\mathrm{Custom}\ \mathrm{o}\mathrm{nly}} = \mathrm{A}*\mathrm{C}\mathrm{o}\mathrm{s}{\mathrm{t}}^{\mathrm{Custom}} + \mathrm{B}*\mathrm{C}\mathrm{o}\mathrm{s}{\mathrm{t}}^{\mathrm{Custom}} $$
- The cost of programmes with ready-made spectacles
  $$ \mathrm{C}\mathrm{o}\mathrm{s}{\mathrm{t}}^{\mathrm{Ready}\hbox{-} \mathrm{made}\ \mathrm{used}} = \mathrm{A}*\mathrm{C}\mathrm{o}\mathrm{s}{\mathrm{t}}^{\mathrm{Custom}} + \mathrm{B}*\mathrm{C}\mathrm{o}\mathrm{s}{\mathrm{t}}^{\mathrm{Ready}\hbox{-} \mathrm{made}} $$
- The cost savings to programmes
  $$ \mathrm{C}\mathrm{o}\mathrm{s}{\mathrm{t}}^{\mathrm{Custom}\ \mathrm{o}\mathrm{nly}}\hbox{-}\ \mathrm{C}\mathrm{o}\mathrm{s}{\mathrm{t}}^{\mathrm{Ready}\hbox{-} \mathrm{made}\ \mathrm{used}} $$

#### Additional analyses

(i) *Reasons for non-spectacle wear*
  Reasons for non-wear will be compared in children who were not wearing ready-made spectacles or custom spectacles.
(ii) *Predictors for spectacle wear*
  We will investigate factors that may affect spectacle wear in this cohort such as gender, age, degree of uncorrected refractive error in the better seeing eye, previously wore spectacles, and parental spectacle wear using multivariable logistic regression analysis.

### Data monitoring

A data monitoring committee will not be required, as both the intervention and comparator arms are not novel procedures and are in common use. There is no reason to expect significant adverse effects. Interim and subgroup analyses are not planned, and there will be no stopping rules.

### Harm

Inaccurate prescribing or fitting of spectacles can cause blurred vision and/or symptoms of eyestrain or headache whilst the spectacles are worn. All refractions in this trial will be undertaken by highly experienced optometrists, and so inaccurate prescribing is highly unlikely. In addition, children who have refractive errors not suitable for ready-made spectacles will not be eligible for the trial, thus reducing the risk of symptoms arising through under/over correction.

Children will not be specifically asked whether they have these symptoms but will be offered the opportunity to say whether symptoms were the reason why they discontinued wearing spectacles at the time of the unannounced follow-up visit. Any child who says that blurred vision, eyestrain or headaches were why they did not wear their spectacles will be refracted again and given a new pair of spectacles, if required.

### Ethics and dissemination

Ethical approval has been obtained from the Interventions Research Ethics Committee, LSHTM and the Institutional Review Board of Sankara Eye Institute. All investigators will contribute to the dissemination strategy, which is likely to include a summary of the findings for head teachers, a report for the website of both institutions, publications in peer-reviewed journals, presentation at national (UK and India) and international conferences.

### Protocol amendment

No important protocol modifications, such as changes to eligibility criteria, were required.

### Consent

Written informed approval has been obtained in the local language by the lead collaborator in India from each school authority, head teacher and/or the school administrator to allow the school to participate in the study. Written informed consent is being obtained from parents of children recruited to the trial. Parents of the children are being sent an information sheet which explains the study procedure along with the consent form in the local language.

Guidelines are being followed for school screening in India and by the collaborating institute which state that before starting screening at each school, children should be given verbal information about the study and an explanation of the procedures by trained field workers, which allows children to ask questions.

### Confidentiality

Data are kept confidential and no identifiers are entered into the databases. Data are anonymised by allocating a unique study ID for each participant. The unique study ID will be used to merge the two study datasets.

Paper records are being stored in a locked filing cabinet at LSHTM, and the data will be made readily available in a public domain after the initial analyses and results are published. At the end of the study, the data will be archived at LSHTM.

### Access to data

A memorandum of understanding has been drawn up between the two institutions highlighting intellectual property issues, which include data sharing and making the database available online.

### Post-trial care

It is recommended that school vision testing be repeated every two years, to identify children whose spectacles need to be replaced as well as to screen children aged 11–12 years for the first time. This will be discussed with head teachers, who may want to consider training teachers to measure visual acuity, with support from Sankara Eye Hospital. This is the process adopted in other schools in the locality.

## Discussion

This trial is designed to investigate whether low-cost, high-quality, ready-made spectacles result in comparable rates of spectacle wear at 3 to 4 months as more expensive custom spectacles and how much cost savings there would be to programmes.

The dissemination strategy will include a summary of the findings for head teachers, a report for the websites of both institutions, publications in peer-reviewed journals and presentations at national (UK and India) and international conferences. In India the findings will be shared with the State Ministry of Health, State Ministry of Education and specifically the Government of India’s ’Rashtriya Bal Swasthya Karyakram’ (RBSK) programme, which includes refractive error, technically called the ‘Child Health Screening and Early Intervention Services’.

### Trial status

At the time of submission recruitment was ongoing. Recruitment started on 12 January 2015 and ended on 31 July 2015. A total of 23,345 children were screened and 460 recruited.

## Abbreviation

D: dioptres
